# Interactions between Thermal Acclimation, Growth Rate, and Phylogeny Influence *Prochlorococcus* Elemental Stoichiometry

**DOI:** 10.1371/journal.pone.0168291

**Published:** 2016-12-09

**Authors:** Adam C. Martiny, Lanying Ma, Céline Mouginot, Jeremy W. Chandler, Erik R. Zinser

**Affiliations:** 1 Department of Earth System Science, University of California, Irvine, California, United States of America; 2 Department of Ecology and Evolutionary Biology, University of California, Irvine, California, United States of America; 3 Department of Microbiology, University of Tennessee, Knoxville, Tennessee, United States of America; Mount Allison University, CANADA

## Abstract

Variability in plankton elemental requirements can be important for global ocean biogeochemistry but we currently have a limited understanding of how ocean temperature influences the plankton C/N/P ratio. Multiple studies have put forward a ‘translation-compensation’ hypothesis to describe the positive relationship between temperature and plankton N/P or C/P as cells should have lower demand for P-rich ribosomes and associated depressed *Q*_*P*_ when growing at higher temperature. However, temperature affects many cellular processes beyond translation with unknown outcomes on cellular elemental composition. In addition, the impact of temperature on growth and elemental composition of phytoplankton is likely modulated by the life history and growth rate of the organism. To test the direct and indirect (via growth rate changes) effect of temperature, we here analyzed the elemental composition and ratios in six strains affiliated with the globally abundant marine Cyanobacteria *Prochlorococcus*. We found that temperature had a significant positive effect on the carbon and nitrogen cell quota, whereas no clear trend was observed for the phosphorus cell quota. The effect on N/P and C/P were marginally significantly positive across *Prochlorococcus*. The elemental composition and ratios of individual strains were also affected but we found complex interactions between the strain identity, temperature, and growth rate in controlling the individual elemental ratios in *Prochlorococcus* and no common trends emerged. Thus, the observations presented here does not support the ‘translation-compensation’ theory and instead suggest unique cellular elemental effects as a result of rising temperature among closely related phytoplankton lineages. Thus, the biodiversity context should be considered when predicting future elemental ratios and how cycles of carbon, nitrogen, and phosphorus may change in a future ocean.

## Introduction

The cellular contents of carbon (C), nitrogen (N), phosphorus (P), and other elements in marine phytoplankton are emerging as important features of ocean biogeochemistry. For a long time, C/N/P was assumed static at Redfield proportions (106/16/1)[1]. However, variability in plankton elemental requirements can influence nutrient limitation patterns and stress [2,3], nitrogen fixation rates [4,5], the link between nutrient supply and C export [6], and atmospheric CO_2_ levels [7]. Recent work has demonstrated extensive differences in the elemental content and ratios of marine communities across regions or seasons [8–12]. However, the exact mechanisms controlling the observed regional differences are still uncertain as key environmental factors strongly co-vary in the ocean.

Multiple biological mechanisms controlling the elemental composition of marine phytoplankton have been proposed. The main suggested controls include nutrient availability, growth rate, temperature, and life history. Extensive experimental and model studies have demonstrated a strong effect of nutrient availability, whereby a low supply of nitrogen or phosphorus leads to a low cell quota (*Q*) of the corresponding element [13–16]. Another important factor is the cellular allocation towards P rich ribosomes at elevated growth rates. Coined the ‘Growth Rate Hypothesis’ [17], fast growth is hypothesized to result in high *Q*_*P*_ and corresponding low C/P and N/P ratios. However, this growth effect on stoichiometry appears to vary extensively by organism and environmental conditions [16,18,19]. Thus, the genetic and environmental contexts (and possible interactions) for changes in growth rate may be important to consider.

Temperature has also been proposed as a relevant factor for setting the elemental allocation in marine phytoplankton but we currently have limited understanding and data for the quantitative effect [20–22]. Toseland and co-workers showed that phytoplankton produce more P-rich ribosomes at lower temperature; putatively to compensate for lower translational efficiency. Hence, temperature was hypothesized to influence the elemental ratios in phytoplankton such that a future warming of the oceans would lead to increasing N/P ratios of marine communities [20]. Supported by a meta-analysis of eukaryotic phytoplankton lineages, Yvon-Durocher and co-workers detected an increase in C/P and N/P (but not C/N) for cells growing at higher temperature [22]. However, temperature affects many cellular processes beyond translation with unknown outcomes on cellular elemental composition. In addition, the impact of temperature on growth and elemental composition of phytoplankton is likely modulated by the life history of the organism. Important life history traits include the thermal growth optimum and more broadly adaptation of individual cellular processes to various temperature conditions. For example, an increase in temperature may have very different physiological effects depending on whether the rise occurs below or above the thermal growth optimum. Thus, the organismal context should be considered for understanding the influence of temperature on the elemental composition of phytoplankton.

The most abundant phytoplankton lineage in the ocean is the marine Cyanobacteria *Prochlorococcus* [23]. The lineage is responsible for a substantial fraction of ocean primary productivity and thus central to ocean biogeochemical functioning. Most studies of phytoplankton elemental stoichiometry are done using eukaryotic lineages with a large cell size that are either rare or absent in the ocean. In contrast, we currently know little about what regulates the elemental composition of *Prochlorococcus* but it appears that changes in growth rate could affect C/N/P [24]. Further, a prior study of *Prochlorococcus* strain MED4 found that concomitant with an increase in growth rate and cell size, C,N, and P quotas increased with temperature, maintaining the same stoichiometry [25]. The *Prochlorococcus* clade also harbors extensive genetic diversity including clades adapted to different ocean temperature regimes [26]. The HLII clade dominates in warm tropical waters, whereas the HLI clade is more common in higher latitude, cooler waters [27]. These distributions are consistent with the growth responses of representative strains, with the HLI strains growing faster than HLII at low temperature, and the HLII strains growing faster than HLI at high temperature. However, it is unknown how adaptations to different ocean regimes and temperature will modulate a thermal effect on the elemental composition.

Here, we investigated the sensitivity of the elemental quotas of *Prochlorococcus* to changes in temperature, with the hypothesis that their N/P and C/P ratios are positively related to temperature. As a possible temperature effect will be modulated by changes in growth rate as well as the life history (i.e., genotype) of the organisms, we quantified the effect of temperature on the growth rate and elemental composition of three strains of the high-temperature-adapted HLII clade and three of the low-temperature-adapted HLI clade. This study contributes fundamental information on how temperature influences the elemental composition of this key, abundant lineage and its contribution to global biogeochemical cycles.

## Materials and Methods

### Strains and growth conditions

Six axenic *Prochlorococcus* strains affiliated with the HLI and HLII clades were analyzed in this study (Table 1). All strains except VOL29 were previously rendered axenic (Table 1), while VOL29 isolation and purification is described presently. VOL29 was isolated during the POWOW1 cruise in the N. Pacific Ocean (29.6°N, 125.07°W) on March 9^th^, 2012 at a depth of 3 m using Instant Ocean Sea Salt media (Spectrum Brands, CA) media to grow under ambient conditions (20–24°C, 40 μmol quanta m^−2^ s^−1^ light) [28]. VOL29 was rendered axenic using the established helper method on agarose plates [29]. A spontaneous streptomycin-resistant derivative was obtained, and plated for colonies on AMP-J agarose medium pre-seeded with the streptomycin-sensitive helper strain EZ55. *Prochlorococcus* colonies were inoculated in AMP-J liquid and then rendered axenic by the addition of streptomycin to eliminate the helper and subsequently verified as axenic [29,30].

**Table 1:** Overview of strains and temperature treatments.

Strain name	Derived from	Clade	Origin	T treatments (°C)	References
**VOL 7**	MED4	HLI	Med Sea	16, 19, 24, 26	[30,45]
**VOL8**	MIT9515	HLI	Eq. Pacific	19, 24, 26	[30,46]
**VOL29**	N/A	HLI	N. Pacific	16, 19, 24	This study
**VOL 4**	MIT9312	HLII	Gulf Stream	19, 24, 26	[30,47]
**VOL1**	MIT9215	HLII	Eq. Pacific	24, 26	[30,48]
**UH18301**	N/A	HLII	N. Pacific	19, 24, 26	[30]

*Prochlorococcus* strains were cultured in filtered (0.2 μm polycarbonate filter, pressure <10 mm Hg) artificial seawater AMP-J medium [29] (per L, 28.1 g NaCl, 6.9 g MgSO_4_*7H_2_O, 5.49 g MgCl_2_*6H2O, 0.67 g KCl. 1.47g CaCl_2_, 0.504 g NaHCO_3_ with 2 ml 0.5 M TAPS, pH 8.0, 1 ml 0.4 M (NH_4_)_2_SO_4_, 2 ml 0.025 M NaH_2_PO_4_ pH7.5, 100 μl 10,000 X Pro99 Trace Metal Mix) with 40 μmol quanta m^−2^ s^−1^ light on a 12:12 light:dark cycle using cool white fluorescent bulbs at temperatures from 16°C to 26°C (Table 1). Cultures were acclimated to the test temperature for at least three transfers (~20 generations) at high cell concentration (> 10^7^cells ml^-1^), before transferring at 10^6^ cells ml^-1^. The purity of strains was tested before and after strains were inoculated to culture. *Prochlorococcus* was inoculated into YTSS and 1/10 ProAC purity test broths in dark and monitored for visible signs of heterotrophic growth [29,30]. Samples were strictly taken during exponential growth. All collected data is listed in S1 Table.

### Cell counting

Concentration of *Prochlorococcus* was measured by flow cytometry using a Guava EasyCyte 8HT cytometer (Millipore, Billerica, MA) and growth rates were estimated.

### Particulate organic matter

Particulate organic carbon (POC), nitrogen (PON) and phosphorus (POP) samples were each collected in duplicate from each of three biological replicates (6 total) by filtration of 50 ml of culture onto precombusted (5 h, 500°C) GF/F filters (Whatman, Florham Park, New Jersey) and stored at -20°C. To quantify POC and PON, filter samples were thawed and allowed to dry overnight at 65°C. Filters were then packed into a 30 mm tin capsule (CE Elantech, Lakewood, New Jersey) and analyzed for C and N content on a FlashEA 1112 nitrogen and carbon analyzer (Thermo Scientific, Waltham, Massachusetts) [31]. POC and PON concentrations were calibrated using known quantities of atropine and peach leaves in each run. The amount of POP was determined in each sample using a modified ash-hydrolysis method [15,32]. We also analyzed multiple blank controls.

### Data analysis

All data was plotted using Matlab. Statistical analyses were done using linear models in R. To account for non-linear effects of T on the elemental content of *Prochlorococcus* strains, T was treated as a factor with four levels.

### Phylogenetic analysis

*Prochlorococcus* ITS nucleotide sequences from each strain were aligned using ClustalW [33]. Pair-wise DNA distance matrix (w. F84 substitution matrix) and neighbor-joining tree were calculated using Phylip v. 3.69 [34] using ITS sequences from *Prochlorococcus* assemblies HNLC1 and HNLC2 as outgroup [35]. Next, we found the linear contribution of temperature, growth rate and strain identity on cell quotas and rates. To evaluate if the strain identity effects were phylogenetically structured, we then compared an Euclidian distance matrix of the strain identity effects to the pair-wise DNA distance matrix using a Mantel test in the R package ‘vegan’ [36].

## Results

**Fig 1:**
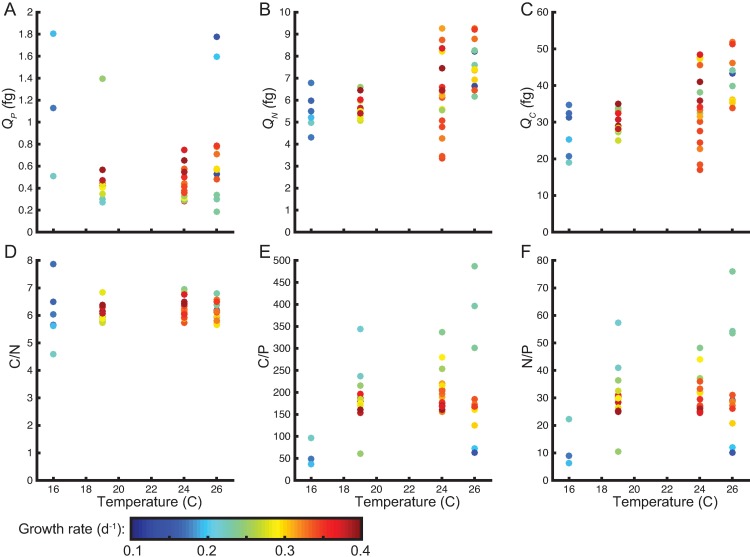
Influence of temperature and growth on the elemental composition and ratios of across *Prochlorococcus* strains. Factors measured are (A) phosphorus cell quota (*Q*_*P*_), (B) nitrogen cell quota (*Q*_*N*_), (C) carbon cell quota (*Q*_*C*_), (D) C/N, (E) C/P, and (F) N/P. The color of each sample point indicates the observed growth rate. All ratios are molar based.

To identify the impact of temperature on the elemental composition of *Prochlorococcus*, we quantified the carbon, nitrogen, and phosphorus cell quota as well as growth rate of six axenic strains (Table 1). HLI and HLII clades, adapted to different temperatures, were represented by 3 strains each, and to facilitate comparisons between clades, the temperatures assayed were within the permissive range for growth of all strains. Median cell quotas across all strains of 0.44 fg P, 6.4 fg N, and 33 fg C were similar to previously measured levels [24,37]. Temperature had a significant linear positive effect on *Q*_*N*_ and *Q*_*C*_ across all strains but no direct effect on *Q*_*P*_ (Table 2 and Fig 1A–1C). Over the 10°C increase in temperature, *Q*_*N*_ and *Q*_*C*_ rose by 40% and 35%, respectively. We also examined the elemental ratios. C/N showed little variability and was close to Redfield proportions (median_C/N_ = 6.1)(Fig 1D). In contrast, C/P and N/P were above Redfield proportions (median_C/P_ = 174, median_N/P_ = 29)(Fig 1E and 1F). Both ratios showed some effect of temperature and there was a marginally significant positive linear trends across all strains (Table 2).

**Table 2:** Effects of temperature, growth rate and strain identity on the elemental composition of six *Prochlorococcus* strains.

		*Q*_*P*_		*Q*_*N*_		*Q*_*C*_		C/N		C/P		N/P	
**Linear model**		Estimate	*p*-value	Estimate	*p*-value	Estimate	*p*-value	Estimate	*p*-value	Estimate	*p*-value	Estimate	*p*-value
	Intercept	1.2	4.9x10^-3^	2.2	0.08	7.8	0.27	5.8	7.6x10^-17^	53	0.6	14	0.3
	Temperature	0.0	1.0	0.2	2.9x10^-4^	1.2	2.3x10^-4^	0.01	0.6	6.8	0.1	0.9	0.1
	Growth rate	-2.0	1.2x10^-2^	-1.6	0.6	-3.3	0.8	0.3	0.8	-65	0.7	-14	0.6
**ANOVA**		SS	*p*-value	SS	*p*-value	SS	*p*-value	SS	*p*-value	SS	*p*-value	SS	*p*-value
	Strain	1.1	0.1	22	1x10^-4^	1x10^3^	6x10^-6^	4.3	7x10^-3^	2x10^5^	2x10^-5^	4x10^3^	1x10^-4^
	Temperature^1^	1.4	2.3x10^-2^	41	5x10^-7^	1x10^3^	8x10^-7^	0.3	0.6	3x10^4^	2.5x10^-2^	6.2x10^2^	4.8x10^-2^
	Growth rate	0.6	3.0x10^-2^	6.5	1x10^-3^	2.8x10^2^	3x10^-4^	0.8	5.3x10^-2^	67	0.9	10	0.7
	Strain:T	0.8	0.5	22	1x10^-3^	5.7x10^2^	3x10^-3^	1.8	0.4	6x10^4^	2.6x10^-2^	1x10^3^	3.9x10^-2^
	Strain:Gr	0.1	0.9	0.9	0.8	28	0.8	0.3	0.9	3x10^3^	0.9	1.x10^2^	0.9
	T:Gr	0.6	0.2	0.8	0.6	6.5	0.9	0.7	0.3	2x10^4^	0.13	5.8x10^2^	5.7x10^-2^
	Strain:T:Gr	0.2	0.9	8.6	7.2x10^-2^	2.4x10^2^	9.6x10^-2^	0.8	0.9	5x10^3^	0.9	89	0.9
**Phylogeny corr.**		R	*p*-value	R	*p*-value	R	*p*-value	R	*p*-value	R	*p*-value	R	*p*-value
	Mantel test	0	0.48	0.19	0.23	0.19	0.23	0.06	0.24	-0.07	0.60	0.02	0.45

^1^ Temperature was treated as factor in ANOVA

**Fig 2:**
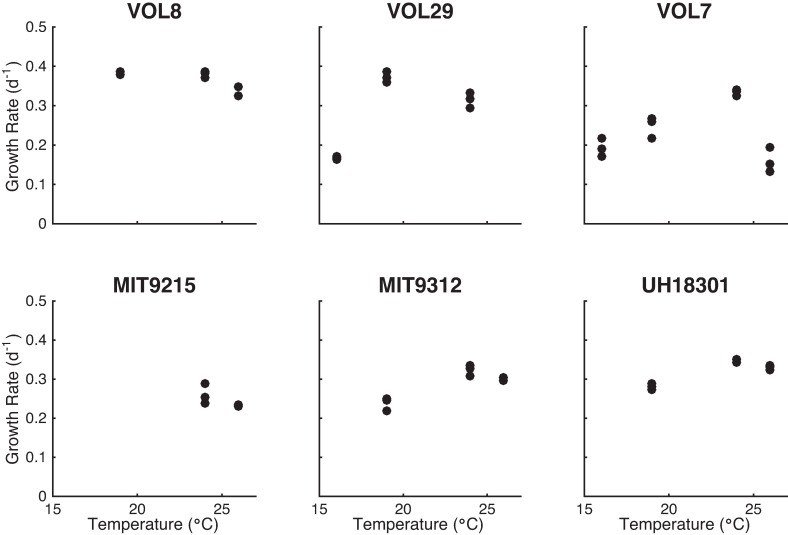
Growth rate of HLI and HLII cultures across a temperature gradient. HLI cultures are VOL8 (MIT9515), VOL29, and VOL7 (MED4) and HLII cultures are MIT9215, MIT9312, and UH18301.

We also quantified growth rates of all the isolates to determine how changes in growth rate in conjunction with temperature affected the elemental composition of *Prochlorococcus* (Fig 2). At a light level of 40 μmol quanta m^−2^ s^−1^, the growth ranged between 0.13 d^-1^ and 0.39 d^-1^. Temperature affected the growth of HLI and HLII isolates slightly different whereby several HLI isolates sustained growth a lower T whereas HLII isolates were less inhibited at high T. Relating growth rate and elemental quotas and ratios, we detected a negative effect of growth rate on *Q*_*P*_, whereas the other cell quotas and ratios did not display any linear trends (Fig 1 and Table 2).

**Fig 3:**
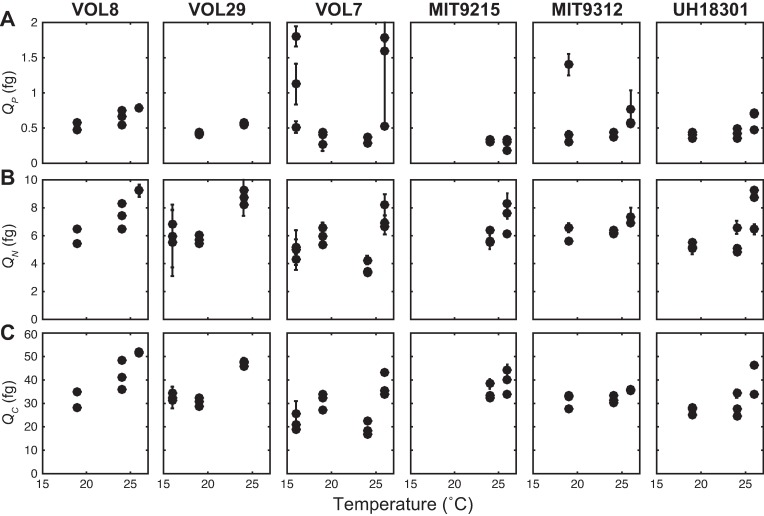
Influence of temperature on cell quotas in six *Prochlorococcus* strains. Cell quotas include (A) phosphorus (*Q*_*P*_), (B) nitrogen (*Q*_*N*_), and (C) carbon (*Q*_*C*_). The error bars represent one standard deviation based on duplicate sampling of each strain.

We next examined the influence of temperature on the cell quotas in the context of each strain as well as indirectly via changes in growth rate (Table 2 and Fig 3). We observed some similarities as well as difference in the response across the six strains. As seen in the aggregated response for all strains, individual strains displayed negative relationships between growth rate and *Q*_*P*_. In addition, temperature also influenced *Q*_*P*_ on a per strain basis (Fig 3A), but there were no systematic differences between strains nor interactions between factors (Table 2). The HLI strains VOL8 and VOL29 had higher overall *Q*_*N*_ and *Q*_*C*_ and temperature plus growth rate influenced *Q*_*N*_ and *Q*_*C*_ across all strains (Fig 3B and 3C). Thus, there was evidence for direct influences of strain identity, temperature, and growth rate–as well as some interactions–in setting the overall elemental composition (Table 2).

**Fig 4:**
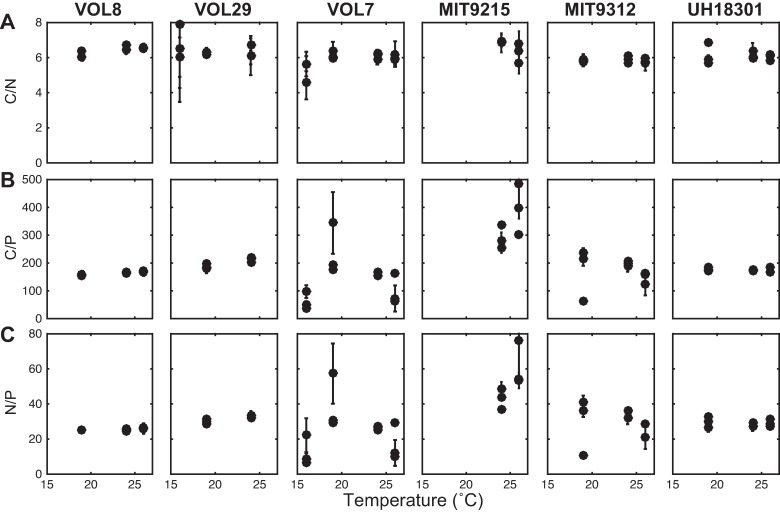
Influence of temperature on elemental ratio in six *Prochlorococcus* strains. Elemental ratios include (A) C/N, (B) C/P, and (C) N/P. All ratios are molar based. The error bars represent one standard deviation based on duplicate sampling of each strain.

Temperature and growth rate also affected the elemental ratios of each strain in unique ways (Fig 4). For C/N, we observed differences in the overall level across the strains, whereby strain VOL8 showed the highest and MIT9312 the lowest level (Fig 4A). The strain specific C/N was also marginally affected by growth rate but not temperature (Table 2). The strain specific C/P and N/P varied considerably between strains (Fig 4B and 4C) and in particular, MIT9215 had considerably higher ratios compared to the other strains. Temperature had a significant impact on C/P and N/P but the direction varied between strains. C/P and N/P in strains VOL8, VOL29, and MIT9215 were positively affected. In contrast, VOL7 showed high variability with lower ratios at 16°C as well as 26°C and higher at the intermediate temperature, UH81301 showed no response, and a negative response was observed in MIT9312. Thus, there were complex interactions between the strain identity and temperature in controlling the elemental ratios in *Prochlorococcus*.

We next found little phylogenetic structuring of elemental changes across strains. After subtracting the overall influence of temperature and growth rates on cell quotas or elemental ratios, we then identified additional variation attributed to each strain. These ‘strain-specific’ contributions were then compared to the phylogenetic distance between each strain (Mantel test, Table 2). This comparison revealed that neither cell quotas nor ratios were phylogenetically structured.

## Discussion

Multiple studies have put forward a ‘translation-compensation’ hypothesis for a positive relationship between temperature vs. N/P or C/P. Cells should have lower demand for P-rich ribosomes and associated depressed *Q*_*P*_ when growing at higher temperature [20,22]. A lower *Q*_*P*_ will cause elevated C/P and N/P and such an acclimation mechanism should further explain the high elemental ratios observed in cells growing in the hot, oligotrophic gyres [8,9]. However, we see little support for this hypothesis in *Prochlorococcus*. Instead, the thermal effect leads to increasing *Q*_*N*_ and *Q*_*C*_, whereas *Q*_*P*_ shows little systematic change. This points towards other physiological acclimation mechanisms as the primary drivers of elemental changes in *Prochlorococcus*. The observed elemental changes are likely associated with a cell size increase as *Q*_*N*_ and *Q*_*C*_ increase in tandem. The underlying mechanism for this increase in *Q*_*N*_ and *Q*_*C*_ in *Prochlorococcus* is not known but the response was opposite to *Scenedesmus* and *Asterionella* [38]. Based on studies in heterotrophic organisms, it is likely associated with an increase in cellular macromolecules and especially protein content [39]. Such a change in cell size means that you cannot simply extrapolate from an increase or a decrease in an individual cell quota (like *Q*_*P*_) to the stoichiometric ratio. Thus, our study adds to an emerging concept, whereby changes in cell size due to physiological responses to different environmental conditions are important for regulating the elemental composition and ratios in marine Cyanobacteria [16].

*Q*_*P*_ appears linked to changes in thermally induced growth rate but not temperature itself. This would indicate support for the growth rate hypothesis [17] but *Q*_*P*_ is actually decreasing at elevated growth rates across all strains as well as for most individual strains. As seen in other marine Cyanobacteria [16], it is clear that the growth rate hypothesis alone cannot explain differences in elemental composition across *Prochlorococcus* strains. However, there is a lot of variation, which suggests individual strain *Q*_*p*_ responses to temperature and growth physiology. The composition of P containing macromolecules underlying the overall cellular P content is poorly constrained in marine Cyanobacteria [15,16] as the sum of phospholipids and nucleic acids does not get close to *Q*_*p*_. Thus, it is currently uncertain, which biochemical mechanism will lead to the observed changes in *Q*_*p*_.

We observe overall high C/P and N/P, whereas C/N is close to Redfield proportions in *Prochlorococcus*. The cells are growing under nutrient replete conditions, which should lead to C/P and N/P at the lower end of the range for an organism [3,40]. Our observations of above Redfield ratios in *Prochlorococcus* are consistent with past observations [8,24,41] and suggest this lineage has overall high C/P and N/P. As such, the presence of *Prochlorococcus* in low latitude marine communities will contribute to elevated elemental ratios independently of thermal and nutrient conditions.

We do not observe a direct phylogenetic structuring of cell quotas and ratios within *Prochlorococcus*. However, we observed a significant influence on the elemental composition via changes in growth rate along the temperature gradient. The temperature effect on growth in *Prochlorococcus* strains have been shown to be strongly phylogenetically structured, whereby the HLI and HLII clades are adapted to lower and higher temperature regimes, respectively [26,27]. Thus, we see an indirect phylogenetic structuring through the effect of growth rate on the cell quotas and ratios. In addition, we see extensive strain variability in the elemental content and ratios due to thermal acclimation. Thus, the organismal context and potentially growth optimum appear important for the individual response. This is consistent with the thermal response in other phytoplankton lineages and strain specific variability in quotas and ratios of *Gyrodinium* species [42,43]. In an analysis across nine eukaryotic phytoplankton lineages, Yvon-Durocher and co-workers observed substantial variability in the link between thermal changes and elemental cellular composition [22]. Furthermore, this meta-analysis as well as our study found little thermal effect on C/N, suggesting C/N being fairly invariant to temperature changes.

The broader environmental growth conditions are important to consider when evaluating the elemental outcome in *Prochlorococcus* to thermal changes. In this study, the cells were growing under nutrient replete conditions and *Prochlorococcus* may store large reserves of P overwhelming any contributions from ribosomal RNA. Multiple studies have shown the possibility for interactions between factors including interactions between nutrient limitation and temperature [22,38]. *Scenedesmus* showed stronger thermal responses under nutrient limited vs. replete conditions. Hence, future work studying the interaction between nutrient limitation and thermal conditions would enhance our understanding for how changes in ocean temperature would affect *Prochlorococcus* elemental stoichiometry.

Our study has implications for understanding both present day and future biogeochemical functioning. The oceans are projected to undergo substantial changes in temperature due to rising CO_2_ in the atmosphere. Such environmental changes will likely have a large impact on phytoplankton community structure and physiology [23,44]. This has been predicted to lead to an increase in N/P ratios in phytoplankton communities [20,22]. However, the observations presented here suggest unique cellular elemental effects as a result of rising temperature among closely related phytoplankton lineages. Thus, the biodiversity context should be considered when predicting future elemental ratios and how the link between the cycles of carbon, nitrogen, and phosphorus may change in a future ocean.

## Supporting Information

S1 Table Data associated with this study. (CSV)

## References

[1] Redfield AC. The biological control of the chemical factors in the environment. Am Sci. 1958;46: 1–18. 24545739

[2] Alexander H, Jenkins BD, Rynearson TA, Dyhrman ST. Metatranscriptome analyses indicate resource partitioning between diatoms in the field. Proc Natl Acad Sci. 2015;112: E2182–E2190. 10.1073/pnas.1421993112 25870299 PMC4418864

[3] Bonachela JA, Allison SD, Martiny AC, Levin SA. A model for variable phytoplankton stoichiometry based on cell protein regulation. Biogeosciences. 2013;10: 4341–4356.

[4] Mills MM, Arrigo KR. Magnitude of oceanic nitrogen fixation influenced by the nutrient uptake ratio of phytoplankton. Nat Geosci. 2010;3: 412–416.

[5] Weber T, Deutsch C. Oceanic nitrogen reservoir regulated by plankton diversity and ocean circulation. Nature. 2012;489: 419–422. 10.1038/nature11357 22996557

[6] Teng Y-C, Primeau FW, Moore JK, Lomas MW, Martiny AC. Global-scale variations of the ratios of carbon to phosphorus in exported marine organic matter. Nat Geosci. 2014;7: 895–898.

[7] Galbraith ED, Martiny AC. A simple nutrient-dependence mechanism for predicting the stoichiometry of marine ecosystems. Proc Natl Acad Sci U S A. 2015;112: 8199–8204. 10.1073/pnas.1423917112 26056296 PMC4500256

[8] Martiny AC, Pham CTA, Primeau FW, Vrugt JA, Moore JK, Levin SA, et al Strong latitudinal patterns in the elemental ratios of marine plankton and organic matter. Nat Geosci. 2013;6: 279–283.

[9] Martiny AC, Vrugt JA, Primeau FW, Lomas MW. Regional variation in the particulate organic carbon to nitrogen ratio in the surface ocean. Global Biogeochem Cycles. 2013;27: 723–731.

[10] Weber TS, Deutsch C. Ocean nutrient ratios governed by plankton biogeography. Nature. 2010;467: 550–554. 10.1038/nature09403 20882009

[11] Martiny AC, Talarmin A, Mouginot C, Lee JA, Huang JS, Gellene AG, et al Biogeochemical interactions control a temporal succession in the elemental composition of marine communities. Limnol Oceanogr. 2016;61: 531–542.

[12] Talarmin A, Lomas MW, Bozec Y, Savoye N, Frigstad H, Karl DM, et al Seasonal and long-term changes in elemental concentrations and ratios of marine particulate organic matter. Global Biogeochem Cycles. 2016; n/a–n/a.

[13] Droop MR. Some thoughts on nutrient limitation in algae. J Phycol. 1973;9: 264–272.

[14] Rhee GY. Effects of N-P atomic ratios and nitrate limitation on algal growth, cell composition, and nitrate uptake. Limnol Oceanogr. 1978;23: 10–25.

[15] Mouginot C, Zimmerman AE, Bonachela JA, Fredricks H, Allison SD, Van Mooy BAS, et al Resource allocation by the marine cyanobacterium *Synechococcus* WH8102 in response to different nutrient supply ratios. Limnol Oceanogr. 2015;60: 1634–1641.

[16] Garcia NS, Bonachela JA, Martiny AC. Interactions between growth-dependent changes in cell size, nutrient supply and cellular elemental stoichiometry of marine *Synechococcus*. ISME J. 2016. 10.1038/ismej.2016.50 PMC5113841 27058506

[17] Sterner RW, Elser JJ. Ecological stoichiometry: the biology of elements from molecules to the biosphere Princeton, NJ: Princeton University Press; 2002.

[18] Zimmerman AE, Allison SD, Martiny AC. Phylogenetic constraints on elemental stoichiometry and resource allocation in heterotrophic marine bacteria. Environ Microbiol. 2014;16: 1398–1410. 10.1111/1462-2920.12329 24237481

[19] Flynn KJ, Raven JA, Rees TA V, Finkel Z, Quigg A, Beardall J. Is the Growth Rate Hypothesis Applicable to Microalgae? J Phycol. 2010;46: 1–12.

[20] Toseland A, Daines SJ, Clark JR, Kirkham A, Strauss J, Uhlig C, et al The impact of temperature on marine phytoplankton resource allocation and metabolism. Nat Clim Chang. 2013;3: 979–984.

[21] Woods HA, Makino W, Cotner JB, Hobbie SE, Harrison JF, Acharya K, et al Temperature and the chemical composition of poikilothermic organisms. Funct Ecol. 2003;17: 237–245.

[22] Yvon-Durocher G, Dossena M, Trimmer M, Woodward G, Allen AP. Temperature and the biogeography of algal stoichiometry. Glob Ecol Biogeogr. 2015;24: 562–570.

[23] Flombaum P, Gallegos JL, Gordillo RA, Rincon J, Zabala LL, Jiao N, et al Present and future global distributions of the marine Cyanobacteria *Prochlorococcus* and *Synechococcus*. Proc Natl Acad Sci U S A. 2013;110: 9824–9829. 10.1073/pnas.1307701110 23703908 PMC3683724

[24] Bertilsson S, Berglund O, Karl DM, Chisholm SW. Elemental composition of marine *Prochlorococcus* and *Synechococcus*: Implications for the ecological stoichiometry of the sea. Limnol Oceanogr. 2003;48: 1721–1731.

[25] Fu FX, Warner ME, Zhang YH, Feng YY, Hutchins DA. Effects of increased temperature and CO2 on photosynthesis, growth, and elemental ratios in marine *Synechococcus* and *Prochlorococcus* (Cyanobacteria). J Phycol. 2007;43: 485–496.

[26] Zinser ER, Johnson ZI, Coe A, Karaca E, Veneziano D, Chisholm SW. Influence of light and temperature on *Prochlorococcus* ecotype distributions in the Atlantic Ocean. Limnol Oceanogr. 2007;52: 2205–2220.

[27] Johnson ZI, Zinser ER, Coe A, McNulty NP, Woodward EM, Chisholm SW. Niche partitioning among *Prochlorococcus* ecotypes along ocean-scale environmental gradients. Science (80-). 2006;311: 1737–1740. 10.1126/science.1118052 16556835

[28] Chandler JW, Lin Y, Gainer PJ, Post AF, Johnson ZI, Zinser ER. Variable but persistent coexistence of Prochlorococcus ecotypes along temperature gradients in the ocean’s surface mixed layer. Environ Microbiol Rep. 2016;8: 272–284. 10.1111/1758-2229.12378 26743532

[29] Morris JJ, Kirkegaard R, Szul MJ, Johnson ZI, Zinser ER. Facilitation of robust growth of *Prochlorococcus* colonies and dilute liquid cultures by “Helper” heterotrophic bacteria. Appl Environ Microbiol. 2008;74: 4530–4534. 10.1128/AEM.02479-07 18502916 PMC2493173

[30] Morris JJ, Johnson ZI, Szul MJ, Keller M, Zinser ER. Dependence of the cyanobacterium *Prochlorococcus* on hydrogen peroxide scavenging microbes for growth at the ocean’s surface. PLoS One. 2011;6: e16805–e16805. 10.1371/journal.pone.0016805 21304826 PMC3033426

[31] Sharp JH. Improved analysis for “particulate” organic carbon and nitrogen from seawater. Limnol Oceanogr. 1974;19: 984–989.

[32] Lomas MW, Burke AL, Lomas DA, Bell DW, Shen C, Dyhrman ST, et al Sargasso Sea phosphorus biogeochemistry: an important role for dissolved organic phosphorus (DOP). Biogeosciences. 2010;7: 695–710.

[33] Larkin M, Blackshields G, Brown N, Chenna R, McGettigan P, McWilliam H, et al ClustalW and ClustalX version 2. Bioinformatics. 2007;23: 2947–2948. 10.1093/bioinformatics/btm404 17846036

[34] Felsenstein J. PHYLIP (Phylogeny Inference Package). 3.65 ed. Seattle: Department of Genome Sciences, University of Washington; 2006.

[35] Rusch DB, Martiny AC, Dupont CL, Halpern AL, Venter JC. Characterization of *Prochlorococcus* clades from iron-depleted oceanic regions. Proc Natl Acad Sci U S A. 2010;107: 16184–16189. 10.1073/pnas.1009513107 20733077 PMC2941326

[36] Oksanen J, Blanchet FG, Kindt R, Legendre P, O’Hara RB, Simpson G, et al. Vegan: Community Ecology Package [Internet]. R package. 2011. Available: http://cran.r-project.org/package=vegan

[37] Heldal M, Scanlan DJ, Norland S, Thingstad F, Mann NH. Elemental composition of single cells of various strains of marine *Prochlorococcus* and *Synechococcus* using X-ray microanalysis. Limnol Oceanogr. 2003;48: 1732–1743.

[38] Rhee G-Y, Gotham IJ. The effect of environmental factors on phytoplankton growth: Temperature and the interactions of temperature with nutrient limitation. Limnology and Oceanography. 1981 pp. 635–648.

[39] Bremer H, Dennis PP. Modulation of chemical composition and other parameters of the cell at different exponential growth rates. EcoSal Plus. American Society of Microbiology; 2008;3. 10.1128/ecosal.5.2.3 26443740

[40] Klausmeier CA, Litchman E, Daufresne T, Levin SA. Optimal nitrogen-to-phosphorus stoichiometry of phytoplankton. Nature. 2004;429: 171–174. 10.1038/nature02454 15141209

[41] Grob C, Ostrowski M, Holland RJ, Heldal M, Norland S, Erichsen ES, et al Elemental composition of natural populations of key microbial groups in Atlantic waters. Environ Microbiol. 2013; 10.1111/1462-2920.12145 23663455

[42] Nielsen M V. Growth and chemical composition of the toxic dinoflagellate Gymnodinium galatheanum in relation to irradiance, temperature and salinity. Mar Ecol Prog Ser. 1996;136: 205–211.

[43] Nielsen M V, Tonseth CP. Temperature and Salinity Effect on Growth and Chemical-Composition of Gyrodinium-Aureolum Hulburt in Culture. J Plankton Res. 1991;13: 389–398.

[44] Boyd PW, Lennartz ST, Glover DM, Doney SC. Biological ramifications of climate-change-mediated oceanic multi-stressors. Nat Clim Chang. 2015;5: 71–79.

[45] Moore LR, Goericke R, Chisholm SW. Comparative physiology of *Synechococcus* and *Prochlorococcus*: influence of light and temperature on growth, pigments, fluorescence and absorptive properties. Mar Ecol Prog Ser. 1995;116: 259–275.

[46] Rocap G, Distel DL, Waterbury JB, Chisholm SW. Resolution of *Prochlorococcus* and *Synechococcus* ecotypes using 16S-23S rDNA internal transcribed spacer (ITS) sequences. Appl Environ Microbiol. 2002;68: 1180–1191. 10.1128/AEM.68.3.1180-1191.2002 11872466 PMC123739

[47] Moore LR, Rocap G, Chisholm SW. Physiology and molecular phylogeny of coexisting *Prochlorococcus* ecotypes. Nature. 1998;393: 464–467. 10.1038/30965 9624000

[48] Moore LR, Chisholm SW. Photophysiology of the Marine Cyanobacterium *Prochlorococcus*: Ecotypic differences among cultured isolates. Limnol Oceanogr. 1999;44: 628–638.

